# Increased Insulin Resistance in Hepatitis-C Infection—Association with Altered Hepatic Function Testing

**DOI:** 10.3390/pathophysiology29030024

**Published:** 2022-06-21

**Authors:** Praveen Raman Mishra, Akash Bharti, Ridhi Arora, Irfan Ahmad Mir, V. P. S. Punia

**Affiliations:** Department of Internal Medicine, School of Medical Science & Research, Sharda University, UP, Greater Noida 201306, India; prmishra83@yahoo.com (P.R.M.); akash.bharti@sharda.ac.in (A.B.); mail.ridhiarora@gmail.com (R.A.); ghnabi5757@gmail.com (V.P.S.P.)

**Keywords:** hepatitis-C, diabetes mellitus, metabolic syndrome, insulin resistance

## Abstract

Introduction: Hepatitis C virus (HCV) infection is a serious global public health problem. It is estimated that 2% to 3% of the world’s population is infected with the virus. It was found that chronic hepatitis C is an independent predictor of the development of type 2 diabetes mellitus. Infection with HCV or the inflammatory response to HCV infection likely contributes to the development of insulin resistance (IR), which increases the risk of developing type 2 diabetes in the long term. This study aimed to assess the insulin resistance in hepatitis C and its correlation with various metabolic parameters. Materials and Methods: This cross-sectional observational study was conducted at a tertiary care hospital in North India in the Department of Internal Medicine with hepatitis C-positive patients attending an out-patient or in-patient department. We took a total of 100 patients aged > 18 years and divided them into two groups: Group A with hepatitis C (cases) and Group B without hepatitis C (controls). There were a total of 50 hepatitis C patients and 50 patients without hepatitis C. Results: A total of 100 patients were included in the present study after obtaining informed consent. There was a significantly higher level of serum ferritin and insulin in group A patients than group B patients. There was a positive correlation of insulin resistance with the serum insulin, ferritin levels, cholesterol, LDL and triglyceride level and a negative correlation with the serum HDL level. The incidence of insulin resistance was positively correlated with changes in fibrosis in the liver due to the hepatitis C infection. Conclusions: From our study, we found that there is an increased incidence of insulin resistance in the patients with hepatitis-C infection, and insulin resistance is associated with the presence of altered hepatic function test results.

## 1. Introduction

Hepatitis C virus (HCV) infection is a serious global public health problem. It is estimated that 2% to 3% of the world’s population is infected with the virus [1,2,3]. It can now be successfully removed due to new therapeutic regimens that are both safe and highly effective [4,5]. Undoubtedly, morbidity and death associated with HCV infection are increasing [6,7]. The results of recent epidemiological studies suggest that chronic HCV infection is an independent predictor of the development of type 2 diabetes mellitus (DM) and that type 2 diabetes is more common in patients with chronic HCV infection than in patients with other causes of liver disease or cirrhosis [8,9,10]. Infection with HCV or the inflammatory response to HCV infection likely contributes to the development of insulin resistance (IR), which increases the risk of developing type 2 diabetes in the long term [11,12,13]. The propensity of insulin resistance to cause progression to fibrosis in chronic hepatitis C patients is the most important adverse consequence of the disease [14,15,16]. There is also evidence that high serum glucose levels are associated with more rapid fibrosis progression, which is greater than obesity. The mean HOMA index increases with fibrosis progression and may be helpful in distinguishing between different stages of fibrosis [17]. This study aimed to assess insulin resistance in hepatitis C infection and its correlation with various metabolic parameters.

## 2. Methods and Materials

From January 2019 to December 2019, this cross-sectional observational study was conducted at a tertiary care hospital in North India in the Department of Internal Medicine with hepatitis C-positive patients attending an out-patient clinic. We took a total of 100 patients aged > 18 years and divided them into two groups. The two groups in the present study comprised 50 hepatitis C (group A) patients and 50 healthy individuals (without hepatitis C) (group B). Informed consent was obtained from all of them. Age, sex, ethnicity, average daily alcohol consumption (g/day) in the past 6 months, average daily alcohol consumption (g/day) before the last 6 months, transfusion history of blood products, waist-to-hip ratio (WHR), body mass index (BMI), intravenous drug use in the past, and the date of a single convincing parenteral exposure were collected at the time of the study (e.g., needle stick injury).

Overnight fasting of 12 h was followed by venous blood draw to assess the serum levels of albumin, bilirubin, alanine aminotransferase (ALT), aspartate aminotransferase (AST), ALT/AST ratio, gamma-glutamyl transferase (GGT), serum ferritin, lipid profile, insulin level, plasma glucose concentration, glycated hemoglobin (HbA1c), platelet count, and the international normalized ratio. The amount of insulin in the blood was measured using a radio-immunoassay. All additional biochemical tests were performed in the clinical biochemistry laboratories of the hospital using automated techniques. The following equation was used to calculate IR using the homeostasis model assessment (HOMA) approach, which yielded the following result:

Insulin resistance (HOMA-IR) = Fasting insulin (U/mL) × Fasting glucose (mmol/L)/22.5. (1)

The antibodies against HCV were tested in all the individuals (Monolisa anti-HCV; Sanofi Diagnostics Pasteur, Marnes-la-Coquette, France) and were later confirmed by viral load. This study was approved by the ethical committee of the institution under reference number SU/SMS&R/76-A/2020/27.

## 3. Statistical Analysis

Using Microsoft Excel, all the data were entered, and statistical analysis was performed using the SPSS v21 software, which was running on Windows 10 (SPSS Inc., Chicago, IL, USA). The information was presented in the form of tables, figures and the data were summarized using the terms mean, standard deviation, frequency, and percentage. A student’s independent t-test was used to compare continuous variables when comparing two groups. When comparing categorical variables, the chi-square test or Fisher’s exact test was used, depending on which was more appropriate. To be considered statistically significant, the two-tailed *p*-value must be less than 0.05.

## 4. Results

A total of 100 patients were included in the present study after obtaining informed consent. The mean age of the participants was 57.65 ± 8.13 with 62 males and 38 females with a male to female ratio of 2:1. The participants were divided into two groups; group A: Hepatitis C infected individuals and group B: Healthy normal controls.

Table 1 below shows the demographic details of the participants. The mean age of the study participants was 55.65 years in group A and 56.01 years in group B, and the distribution of males was 62 and females was 38, with a male preponderance in the present study. The table also shows that the BMI of the hepatitis C group was more than normal controls with a *p*-value of <0.001.

Table 2 shows us the mean values of various parameters in both groups in which there was a statistically significant difference between liver enzymes. The blood cholesterol, triglyceride, TG/HDL ratio (triglyceride and HDL ratio) and LDL cholesterol in group A were significantly higher than in group B. However, when comparing the patients in group A to the patients in group B, the serum HDL cholesterol was considerably lower in group A.

From Table 3 we can find a statistically significant difference between mean levels of serum ferritin, serum insulin and insulin resistance between the two groups.

Table 4 shows a statistically significant positive correlation of the relationship between HbA1c, insulin resistance, serum levels of insulin, and ferritin in this study. Based on these findings, it appears that hepatitis C infection is associated insulin resistance and an increase in inflammatory markers such as ferritin, in the patients studied.

The above figure shows a significantly higher number of patients with fibrosis also having the presence of insulin resistance.

## 5. Discussion

Liver fibrosis has been considered for a long time to be responsible for the appearance of insulin resistance in patients with chronic liver diseases [18]. Simultaneous measurements of C-peptide and insulin revealed that both insulin resistance and insulin secretion contribute to glucose intolerance in patients with chronic HCV [19]. However, there is a suggestion that the mechanism of hepatic steatosis underlying HCV infection might differ from that of NAFLD and should be explored [20].

From our study results, we found that hepatitis C infection is associated with the presence of insulin resistance and an increase in inflammatory markers such as ferritin. As shown in Table 3, the serum levels of ferritin and insulin were significantly higher in group A than the group B. Similarly, the serum insulin resistance calculated based on the formula was found to be significantly higher in group A (5.91 ± 1.21) than in group B (3.55 ± 1.17) with *p* < 0.001. These findings go in accordance with the study done by Ray et al. [21], Laloo et al. [22] and Hum et al. [23]. Insulin resistance is associated with hepatitis C, which has been considered by several different researchers to be an important finding [24,25].

**Figure 1:**
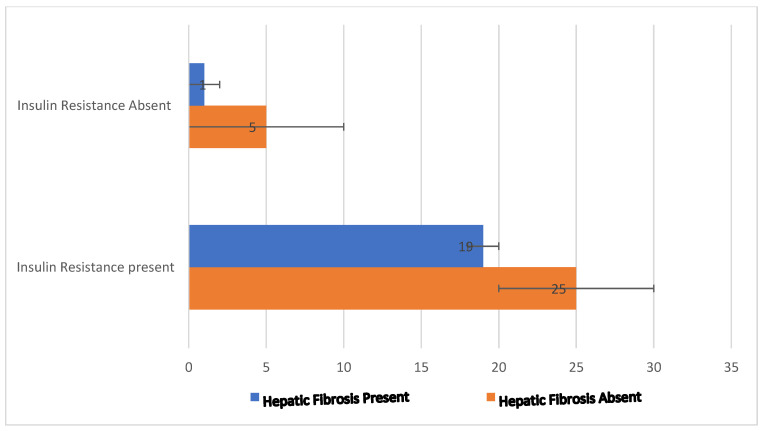
Showing the presence of insulin resistance among patients with hepatic fibrosis.

From Figure 1, we found a significantly higher number of patients with fibrosis showing the presence of insulin resistance. There is a complex relationship between the presence of the hepatitis C infection with the development of insulin resistance [26,27]. Available studies have indicated that HCV core protein expression induces hepatic insulin resistance by altering the signaling pathway of the insulin receptor substrate-1 [28,29]. This can result in the expression of the diabetic phenotype, along with other factors such as diet and obesity. When insulin resistance reaches the extent that the β-cell no longer compensates, insulin secretion decreases, and hyperglycemia develops [30,31,32]. The complex relationship with the host hepatic glucose and lipid metabolism of chronic HCV infection is not fully understood and remains to be determined.

From Table 1, we also found that systolic blood pressure and diastolic blood pressure were both substantially higher in group A patients (*p* = 0.05). A greater level of blood pressure in hepatitis C patients predicts a higher risk of coronary artery disease [33].

From Table 2, the assessment of liver function revealed that the members in group A had aberrant results, which were statistically significant. The total protein and albumin levels in the serum of group A were considerably lower than those in the control group. The detoxifying functions of the liver, which include the level of bilirubin, were found to be considerably higher in group A than in the other groups. In group A, the enzyme levels AST and ALT were borderline, but significantly higher than in the group B patients. These data indicate that there was some liver injury in the body. These findings are consistent with a study done by Naing et al. [24]. The hematological parameters in cases were found to be not significantly different from those of the control group. Blood glucose levels measured before meals as well as after meals were not substantially different between the two groups of patients. Both groups of individuals were found to have diabetes control that was in the middle of the range at different points in time. Mohammed et al. describe findings that are similar [25].

Although from Table 2, there were no statistically significant differences in the lipid profiles of the patients in group A vs. group B. The blood cholesterol, triglyceride, and LDL cholesterol in group A were significantly higher than in group B. The triglyceride: HDL ratio in group A was higher than in group B which also indicates more insulin resistance in group A, and according to a study conducted by Borrayo et al. [34] increased triglyceride and HDL ratio is associated with more insulin resistance. However, when comparing the patients in group A to the patients in group B, the serum HDL cholesterol was considerably lower in group A. This agrees with the study done by Felmlee et al. [35].

## 6. Limitations

The major limitation of our study is that it was a cross-sectional study, and we could not find any evidence of a temporal relationship between exposure and outcome. Also, the size of the sample was small.

## 7. Conclusions

From our study, we found that there is an increased incidence of insulin resistance in patients with hepatitis-C infection, and insulin resistance is associated with the presence of altered hepatic function test results. There is also an association of insulin resistance with an increase in LDL cholesterol and a decrease in HDL cholesterol level. Insulin resistance is also correlated with inflammatory markers like ferritin.

## Figures and Tables

**Table 1:** Demographic details of the patients in both groups.

	Group A		Group B		*p*-Value
	Mean	SD	Mean	SD	
Age in years	55.65	7.13	56.01	7.51	0.71
BMI in kg/m^2^	29.43	1.76	24.22	1.91	<0.001
Waist circumference in centimetres	99.05	6.86	95.05	7.92	0.05
Systolic blood pressure	146.62	14.60	129.5	11.1	0.05
Diastolic blood pressure	92.76	8.57	86.01	7.85	0.05
Duration of diabetes mellitus in years	4.40	3.51	4.2	3.40	0.772

* *p*-value < 0.05 is statistically significant.

**Table 2:** Mean levels of the various biochemical parameters in both groups.

		Group A		Group B		*p*-Value
		Mean	SD	Mean	SD	
Hemogram	Haemoglobin gm%	13.90	0.95	14.01	1.01	0.61
	Total count	8283.82	471.79	7912.11	419.2	0.001
	MCV	94.40	3.96	92.01	4.01	0.012
	MCHC	31.50	5.30	32.5	5.9	0.18
Blood glucose	Fasting blood glucose in mg/dL	148.69	34.19	151.11	36.35	0.66
	Post prandial blood glucose in mg/dl	230.21	64.33	250.01	66.29	0.12
Lipid profile	Total cholesterol in mg/dL	209.82	45.08	199.1	35.85	0.19
	Triglyceride in mg/dL	161.86	27.11	159.56	30.17	0.72
	HDL cholesterol in mg/dL	38.87	5.69	40.95	6.01	0.05 *
	Triglyceride/HDL ratio	4.16	1.23	3.9	1.07	0.05 *
	LDL cholesterol in mg/dL	128.59	32.52	130.24	30.43	0.74
Liver Function test	Total protein	6.52	1.58	7.01	1.34	0.05 *
	Albumin	3.1	1.01	3.98	0.91	0.05 *
	Total bilirubin	1.9	0.58	0.8	0.34	0.05 *
	Direct bilirubin	0.65	0.21	0.21	0.09	0.05 *
	Indirect bilirubin	1.25	0.89	0.59	0.22	0.05 *
	AST	41	5.68	28	2.98	0.05 *
	ALT	49	6.45	26	2.55	0.05 *
Renal Function test	Urea in mg/dL	32.38	9.110	33.01	5.91	0.68
	Creatinine in mg/dL	1.0343	0.32381	0.98	0.21	0.357

* *p*-value < 0.05 is statistically significant.

**Table 3:** Showing the mean levels of ferritin, serum insulin and insulin resistance in both groups.

	Group A		Group B		*p*-Value
	Mean	SD	Mean	SD	
Ferritin in ng/mL	324.04	53.112	162.17	42.01	0.001
Insulin	4.91	2.29	3.87	2.07	0.02
Insulin resistance	5.91	1.21	3.55	1.17	0.001

* *p*-value < 0.05 is statistically significant.

**Table 4:** Pearson’s correlation of insulin resistance with other parameters.

		Glycated Hemoglobin (HbA1c)	Insulin	Insulin Resistance	Ferritin
Glycated hemoglobin (HbA1c)	r	1	−0.130	0.094	−0.185
	Sig		0.192	0.350	0.063
Insulin	r	−0.130	1	0.960 **	0.546 **
	Sig	0.192		0.000	0.000
Insulin resistance	r	0.094	0.960 **	1	0.512 **
	Sig	0.350	0.000		0.000
Ferritin	r	−0.185	0.546 **	0.512 **	1
	Sig	0.063	0.000	0.000	

** Correlation is significant at the 0.01 level (2-tailed).

## Data Availability

Not applicable.
